# Hepatitis B Virus / Human Immunodeficiency Virus Co-Infection and Its Hepatocarcinogenic Potential in Sub-Saharan Black Africans

**DOI:** 10.5812/hepatmon.7876

**Published:** 2012-10-30

**Authors:** Michael C. Kew

**Affiliations:** 1 Department of Medicine, Johannesburg Academic Hospital, University of the Witwatersrand, Johannesburg, South Africa; 2 Department of Medicine, Groote Schuur Hospital, University of Cape Town, Cape Town, South Africa

**Keywords:** Hepatitis B virus, Carcinoma, Hepatocellular, Tumor

## Abstract

**Context:**

Since the introduction of highly active anti-retroviral regimen for human immunodeficiency virus-1 infection, a significant increase in the incidence of hepatocellular carcinoma has been reported in patients already chronically infected with hepatitis B virus and then given this form of regimen for their retroviral infection.

**Evidence Acquisition:**

This phenomenon was initially attributed to the far more prolonged survival of those patients who received this new regimen, which provided sufficient time, allowing hepatitis B virus-induced hepatocellular carcinoma to develop.

**Results:**

The current belief is that the increased incidence of hepatocellular carcinoma is because of co-infection with the two viruses, one known to be hepatocarcinogenic and the other suspected to increase the carcinogenic potential of the other. Because both hepatitis B virus and human immunodeficiency virus -1 are endemic in the Black population of sub-Saharan Africa and are transmitted in similar ways, as many as 20% of this population are co-infected with the two viruses. In this way, the already high risk of Black African patients developing hepatitis B virus-induced hepatocellular carcinoma is further increased.

**Conclusions:**

The pathogenetic mechanism or mechanisms involved in the carcinogenic interaction between the hepatitis B virus and the human immunodeficiency virus-1 in sub-Saharan Black Africans and other populations co-infected with these viruses have yet to be determined.

## 1. Context

### 1.1. Hepatocellular Carcinoma

Based on the number of new cases of cancer in humans each year, hepatocellular carcinoma (HCC) is the sixth most common cancer worldwide, the fifth in males and the seventh in females (1). While the number of new cases of the tumor has been recorded, this number has been increased every year. In the most recent reported year (2008), 748,000 new cases of HCC were recorded (1), constituting 9.2% of all new cancers (2), 522,000 in males and 226,000 in females (1). Of these cases 626,700 occurred in resource-constrained (developing) regions, 440,700 in males and 186,000 in females (1). These data confirm previous information in which approximately 83% of global HCCs have been occurred in resource-constrained regions (2, 3). The incidence of the tumor differs from high-risk and low-risk regions of HCC by as much as 100-fold, a ratio which is amongst the highest for any of the major human cancers. Published incidences of HCC in sub-Saharan Africa, a resource-constrained region, underestimate the true incidence of the tumor because of many instances in which HCC is not either definitively diagnosed or recorded in any cancer registry. Nevertheless, at least 46,000 new cases of HCC are known to occur each year (4), with incidences as high as 101.7 per 100,000 persons/year in males and 31.4 per 100,000 persons/year in females being recorded in Mocambique in southern Africa (5), which is the highest reported incidence of the tumor ever recorded in the world. HCC also carries an especially grave prognosis. In the year mentioned, the global death rate from the tumor was 695,900 (1). Of these deaths, 478,300 were in males (the second most common cause of death from cancer), and 217,000 in females (the sixth most common cause of death from cancer) (1). Additionally the prognosis of HCC is especially grave in resource-constrained geographical regions, including sub-Saharan Africa. During the same year, the number of deaths in these regions was 580,600, in which 402,900 occurred in males (the second most common cause of cancer deaths) and 177,000 in females (the fifth most common cause of cancer deaths) (1). The 12-month fatality ratio for HCC (0.94) in resource-constrained regions, including that in sub-Saharan Africa, is the highest of any human tumor (1).

## 2. Evidence Acquisition

### 2.1. Chronic Hepatitis B Virus Infection

Hepatitis B virus (HBV) infection is today the leading cause of chronic liver diseases and liver-related deaths worldwide, with approximately 400 million people chronically infected with the virus and at risk of developing the life-threatening complications of cirrhosis and HCC (3). Infection with this virus is the tenth leading cause of deaths worldwide, accounting for as many as 1.4 million deaths each year (6, 7). Approximately 45% of the world’s population live in areas endemic for HBV, and are thus at high risk of becoming infected with this virus. HBV infection is thus a major public health threat, being on a similar scale of magnitude to human immunodeficiency virus infection (HIV-1), malaria, and tuberculosis (8). Approximately 65 million people live in sub-Saharan Africa, which the most are Blacks and live in rural areas. Under-reporting and inefficient collection of data make it difficult to accurately quantitate the burden of HBV infection in the sub-continent. Despite this, sub-Saharan Africa is known to have the highest incidence of HBV endemicity in the world (9). In the Black African population, as in other high prevalence populations, more than 8% (and as many as 15%) of the population are chronic carriers of the HBV, and between 70% and 98% show serological evidence of being exposed to the virus (6, 10, 11). Both exposure and carrier rates are higher in Black African rural than urban dwellers (12, 13). Chronic HBV infection may be the cause of as much as 84% of HCCs in sub-Saharan African Black Africans (13-16).

### 2.2. Human Immunodeficiency Virus Infection

Between 33 and 40 million people worldwide are infected with the human immunodeficiency virus -1 (HIV-1), with approximately 2.7 million new cases and two million deaths occurring each year (17, 18). Because of the high frequency of co-infection of HIV and HBV in Africa and Asia, HIV-1 infection alone is more common in resource-rich regions. With the increasing availability of highly active anti-retroviral regimen (HAART), there has been a significant decrease in the incidence of opportunistic HIV-1 infections, hospitalizations and in-patient costs, with a precipitously fewer AIDS associated deaths and a corresponding more people surviving with HIV-1 infection (17-19). One of the consequences of this transition has been the emergence of liver diseases as a major cause of death in HIV-1 infected persons (20-22).

HIV-1 infection may result in abnormal liver function tests, hepatic fibrosis, liver decompensation with or without histological evidence of cirrhosis, non-alcoholic steatohepatitis and its more severe forms, non-alcoholic steatohepatitis, and HCC (23-25). The virus may infect multiple cells in the liver, resulting in enhanced intrahepatic apoptosis and fibrosis. HIV-1 may also alter gastrointestinal tract permeability, leading to increased levels of circulating lipopolysaccharide that may have an impact on liver function (23-25). In a large Swiss cohort of individuals infected with HIV-1 alone, 16% had chronically elevated alanine aminotransferase levels (23). Most individuals infected with HIV-1 live in sub-Saharan Africa or Asia. Those living in sub-Saharan Africa constitute 67% of the global burden of the virus, with 22.4 million HIV-1-infected people living in the sub-continent (26-29). The overall incidence of the infection is 11%, although incidences as high as 21% have been recorded in Lesotho and 38.8% in Botswana, both southern African countries (26-29).

### 2.3. Human Immunodeficiency Virus / Hepatitis B Virus Co-Infection

HBV and HIV-1 individually are among the 10 leading causes of infectious diseases deaths worldwide (30, 31). The two viruses are transmitted in similar ways and 5% to 15% of individuals worldwide who are infected with HIV are also infected with HBV (17-19, 26, 30-34). In countries in which HIV-1 is highly endemic, association rate between HIV-1 and HBV may be as high as 25%. Of the individuals infected with HIV-1, two to four million are estimated to be chronically co-infected with HBV (35). Patients co-infected with HBV and HIV-1 have increased rates of HBV replication and progression to chronic hepatitis and cirrhosis more quickly and more often than those with HBV alone, and are therefore at greater risk of developing HCC (30). Moreover, the liver-related mortality rate from combined HBV and HIV-1 infections is increased beyond each infection alone: 14.2/100,000 in those with combined infection, compared to 1.7/100,000 in those with HIV infection alone or 0.8/100,000 in those with HBV infection alone (17). The impact of co-infection is especially apparent in regions with widespread use of HAART. In co-infected individuals the liver-related mortality rate was highest with those with lower nadir CD4+ cell counts (17).

Among HIV-1-infected persons in areas of low HBV endemicity in Western Europe and the United States, co-infection is usually acquired in adulthood because of unsafe sexual practices and, to a lesser extent, drug abusers injection (20, 22, 23, 35, 36). Between 6% and 14% of these individuals at risk are chronically infected with HBV, including 4% to 6% % of HIV-1-positive heterosexuals, 9% to 17% of men who have sex with men, and 7% to 10% of injection drug users (30). In these circumstances HBV is 100 times more infectious than HIV-1. In another study the increased risk of HCC among Swiss men co-infected with HIV-1 and HBV and with low CD4+ cell counts, who have had sex with men, heterosexual sex, or other sex, was greater than that in injection drug users (36). As a consequence of these modes of transmission, HBV/HIV-1 infection affects essentially the adult population.

Co-infections with HBV and HIV-1 are common, not only due to shared modes of transmission of the viruses, but also because HIV-1 infection causes multi-dimensional immune suppression, which reduces the probability of spontaneous recovery from HBV infection (17). Liver disease is now the leading cause of morbidity and mortality in individuals co-infected with the two viruses (31, 37). In the era of widespread use of HAART, the mortality of AIDS-defining diseases has been dramatically declined (17). For example, the number of deaths among HIV-positive individuals in 1996 was 7/100,000, but this figure had been already declined to 1/100,000 individuals by 2004 (17). But, during this time proportional increases in liver diseases, partly associated with HBV infection and partly with hepatitis C virus (HCV) infection, emerged as a major cause of morbidity and mortality in HIV-1-infected persons (22, 23), with a mortality rate twice as high as that in the pre-HAART era (18).

Since the introduction of HAART for the treatment of HIV-1/acquired immunodeficiency syndrome (HIV/AIDS), more patients co-infected with HIV-1 and HBV and receiving this regimen have been reported to develop HCC (26, 31-34, 36-40). Consequently, this tumor has become an increasingly frequent cause of death in patients co-infected with HBV/HIV-1, to the point that it is now the major cause of death in these patients. The mortality rate is currently twice that in 1996 (18), with a 36% excess risk of all-causes mortality (26, 31-34, 36-40). An improved life expectancy in the HAART era might provide a longer time for cirrhosis to develop, and also for both HAART-associated hepatic toxicity (25, 40) and immune restoration to occur (22, 32). Studies undertaken in Western countries have shown that individuals co-infected with HIV-1 and HBV are more likely to be HBeAg-positive, have higher levels of HBV DNA in their sera, and have lower serum alanine aminotransferase concentrations compared to individuals without HIV-1 infection, suggesting that the immune response to HBV in these individuals is less vigorous (18, 33, 34). Any deleterious effect arising from the difference between the HBeAg-positivity rate alone would, however, be less likely to be a factor in sub-Saharan Black African patients with HIV-1/HBV because HBeAg is known to seroconvert to anti-HBe positivity at a significantly earlier age in this population (11, 41).

### 2.4. Human Immunodeficiency Virus /Hepatitis B Virus Co-Infection in Sub-Saharan Black Africans

Both HBV and HIV are endemic, or even hyperendemic, in the Black population of sub-Saharan Africa, with as many as 20% of the population living in the sub-continent being co-infected with the two viruses (26-29, 42-46). In the only study conducted in the sub-continent in which occult HBV infection has been evaluated in patients with HBV/HIV-1 co-infection, the number of patients co-infected with HBV and HIV-1 increased from 4.8% without testing to 12.4% with testing (45). As HAART continues to be increasingly introduced in a large scale into sub-Saharan African countries, it is likely that the consequent HBV/HIV-1 induced HCC will become as an even greater complication (27). In sub-Saharan Black Africans, the way in which HBV is transmitted among individuals and the timing of the infection differs from what is occurring in economically-rich countries. In Black Africans the infection is predominantly acquired before the age of 5 years. In a relatively small percentage of those infected (less than 1 %), HBV infection is acquired because of the perinatal transmission of the virus from a highly infectious HBV-positive carrier mother to her babies (46). In most of infants or young children infected, the infection is acquired a little later because of the ‘horizontal’ transmission of the virus (47). The source of this infection is predominantly an infected mother or a recently infected young sibling or playmate, nevertheless infected fathers provide a lower risk. In rural areas, the virus may be transmitted, in addition to ritual tattooing, incision, or scarification of the skin, or minor ritual surgical procedures, such as circumcision or amputation of the terminal phalanx of the little finger, performed by the local traditional healer (witch-doctor, “inyanga”) using instruments which have been never sterilized and in ceremonies involving a number of infants or very young children from the village. Detection of HBV in blood-sucking insects, such as mosquitoes and bed bugs, has suggested the probable transmission risk of the virus from insects’ bites to humans; furthermore this source has to be proven. In sub-Saharan Black African adults who acquire the infection, the major route of transmission of the virus is heterosexual practices, rather than the homosexual ones . More than 95% of adults in all geographical regions, Including sub-Saharan Africa, spontaneously recover from an acute HBV infection (17).

### 2.5. Hepatitis B Virus/Hiv Co-Infection as a Possible Cause for Hepatocellular Carcinoma With Emphasis on the Occurrence in Sub-Saharan Black Africans

Until the latter half of the 1990s, no increase in the number of patients infected with HIV-1 developing HCC was reported. Patients were, surviving for a short period of time which was unlikely that sufficient time to allow the tumor to develop. But with the increasing occurrence of HCC in patients infected with HIV-1 after the introduction and widespread use of HAART for the treatment of the retroviral infection, among the possible explanations was the prolonged survival of patients with HIV-1 infection now possible with HAART in comparison with the earlier short survival time, might be complicated by hepatocellular carcinogenesis. Two published studies addressed the question of the hepatocarcinogenic potential of HIV-1 infection alone. The first, performed in Black patients in southern Africa, showed that HIV-1 infection alone does not cause HCC (48), and this finding was confirmed in a later considerably larger study performed in the United States (49). Another possible explanation for the increasing incidence of HCC in HIV-1-positive patients was the far longer survival of the patients because HAART provided sufficient time for risk factors known to cause HCC, notably chronic hepatitis C virus or HBV infection, to be responsible for the development of the tumor (50, 51). Although this possible explanation held sway for a short time, it soon became apparent that the increasing incidence of the tumor was because of an increasing incidence of patients with HCC who were co-infected with either HIV-1 and HCV or HIV-1 and HBV. Chronic HBV infection is universally recognised as a major cause of HCC. Subsequent to the introduction and widespread use of HAART, between 5% and 15% of all HIV-1-positive patients with HCC have been reported to be co-infected with HBV (26-38, 40, 42). Almost all of the remaining patients have some clues of previous HBV infection, especially in sub-Saharan Black Africans who are almost always exposed to this virus in early childhood (47). This observation is, however, unlikely to be relevant when considering the pathogenesis of the malignant transformation. Although a global increase in incidence of HCC has been occurred over this period (1-4), a parallel increase specifically attributed to HBV infection alone has not been documented. In an attempt to exclude a role for more prolonged survival due to the treatment with HAART and also to confirm whether a hepatocarcinogenic interaction does occur between the two viruses, a retrospective comparison of the occurrence of HCC in patients co-infected with HIV-1 and HBV before and after the introduction of HAART would be of limited use because of the very short survival time of the patients in the pre-HAART era. Furthermore, a prospective study of the development of the tumor in patients with dual HBV/HIV-1 infection receiving or not receiving HAART is ethically unacceptable. A case-control comparing the incidence of HBV/HIV-1 co-infection in the serum of patients with HBV-induced HCC with that in matched asymptomatic carriers of HBV, both groups from the pre-HAART era, might be a pointer to an increased hepatocarcinogenic potential of the two viruses in tandem. One such study in southern African Blacks showed a statistically significant higher incidence of HBV/HIV-1 co-infection in HCC patients than in controls (52). But the study had limitations prevented reaching a firm conclusion. 

## 3. Results

Two possible ways in which HIV-1 co-infection might indirectly increase the risk of HBV-induced HCC should be taken into account. High HBV loads are known to increase the risk of HCC development (53, 54). Immune suppression of the host induced by HIV-1 infection could result in higher HB viral loads, thereby increasing the risk of HBV-induced hepatocellular carcinogenesis. HIV-1-positive individuals co-infected with HBV in adulthood have been shown to have an increased rate of HBV replication, and progression to chronic hepatitis five times more than HIV-negative individuals, thereby accelerating progression to cirrhosis and hence to HCC (17, 26, 55).

## 4. Conclusions

Very little laboratory research directed at proving or disproving a synergistic hepatocarcinogenic interaction between HBV and HIV-1, or unveiling possible mechanisms of an interaction between the two viruses in the pathogenesis of HCC, has thus far been published, however there are undoubtedly considerable investigations on the subject in progress. The HBV x gene and X protein are known to play a key role in the pathogenesis of HCC induced by this virus (41), and HBV X protein has been shown to induce HIV-1 replication and transcription in synergy with Tat protein and T-cell activation signals (56). Moreover, observations in transgenic mice in which HIV Tat protein had been expressed constitutively in the liver, enhanced the effect of a number of hepatocarcinogens (57) and results in a high incidence of HCC after a long latency (58). It is hoped that the results of the current and future researches at a molecular level will provide one or more explanations for the synergistic hepatocarcinogenic interaction between HBV and HIV-1.
